# Discontinuation of psychotropic medication: a synthesis of evidence across medication classes

**DOI:** 10.1038/s41380-024-02445-4

**Published:** 2024-03-19

**Authors:** Christiaan H. Vinkers, Ralph W. Kupka, Brenda W. Penninx, Henricus G. Ruhé, Jakob M. van Gaalen, Paul C. F. van Haaren, Arnt F. A. Schellekens, Sameer Jauhar, Josep A. Ramos-Quiroga, Eduard Vieta, Jari Tiihonen, Stijn E. Veldman, Wim Veling, Roeland Vis, Laura E. de Wit, Jurjen J. Luykx

**Affiliations:** 1 Department of Psychiatry and Anatomy & Neurosciences, Amsterdam University Medical Center location Vrije Universiteit Amsterdam, 1081 HV Amsterdam, The Netherlands; 2 Amsterdam Public Health, Mental Health Program and Amsterdam Neuroscience, Mood, Anxiety, Psychosis, Sleep & Stress Program, Amsterdam, The Netherlands; 3 GGZ inGeest Mental Health Care, Amsterdam, The Netherlands; 4 Department of Psychiatry, Amsterdam Neuroscience and Amsterdam Public Health, Amsterdam UMC, Vrije Universiteit Amsterdam, Amsterdam, The Netherlands; 5 Department of Psychiatry, Radboudumc, Radboud University, Nijmegen, The Netherlands; 6 Donders Institute for Brain, Cognition and Behavior, Radboud University, Nijmegen, The Netherlands; 7 Nijmegen Institute for Scientist Practitioners in Addiction (NISPA), Nijmegen, The Netherlands; 8 Centre for Affective Disorders, Psychological Medicine, IoPPN, King’s College, London, UK; 9 Department of Mental Health, Hospital Universitari Vall d’Hebron, Barcelona, Catalonia, Spain; 10 Group of Psychiatry, Mental Health and Addictions, Vall d’Hebron Research Institute (VHIR), Barcelona, Catalonia, Spain; 11 Biomedical Network Research Centre on Mental Health (CIBERSAM), Barcelona, Catalonia, Spain; 12 Department of Psychiatry and Forensic Medicine, Universitat Autònoma de Barcelona, Barcelona, Catalonia, Spain; 13 Bipolar and Depressive Disorders Unit, Hospital Clinic, Institute of Neuroscience, University of Barcelona, IDIBAPS, CIBERSAM, Barcelona, Catalonia, Spain; 14 Department of Forensic Psychiatry, Niuvanniemi Hospital, University of Eastern Finland, Kuopio, Finland; 15 Department of Clinical Neuroscience, Karolinska Institutet, 11364 Stockholm, Sweden; 16 Center for Psychiatry Research, Stockholm City Council, Stockholm, Sweden; 17 Neuroscience Center, University of Helsinki, Helsinki, Finland; 18 Novadic-Kentron Addiction Care, Vught, The Netherlands; 19 Department of Psychiatry, University Medical Centre Groningen, University of Groningen, Groningen, The Netherlands; 20 Department of Clinical Pharmacy, St. Antonius Hospital, Nieuwegein/Utrecht, The Netherlands; 21 Department of Psychiatry, St. Antonius Hospital, Nieuwegein/Utrecht, The Netherlands; 22 Department of Psychiatry and Neuropsychology, School for Mental Health and Neuroscience, Maastricht University Medical Centre, Maastricht, The Netherlands

**Keywords:** Addiction, ADHD, Bipolar disorder, Depression, Schizophrenia

## Abstract

Pharmacotherapy is an effective treatment modality across psychiatric disorders. Nevertheless, many patients discontinue their medication at some point. Evidence-based guidance for patients, clinicians, and policymakers on rational discontinuation strategies is vital to enable the best, personalized treatment for any given patient. Nonetheless, there is a scarcity of guidelines on discontinuation strategies. In this perspective, we therefore summarize and critically appraise the evidence on discontinuation of six major psychotropic medication classes: antidepressants, antipsychotics, benzodiazepines, mood stabilizers, opioids, and stimulants. For each medication class, a wide range of topics pertaining to each of the following questions are discussed: (1) Who can discontinue (e.g., what are risk factors for relapse?); (2) When to discontinue (e.g., after 1 year or several years of antidepressant use?); and (3) How to discontinue (e.g., what’s the efficacy of dose reduction compared to full cessation and interventions to mitigate relapse risk?). We thus highlight how comparing the evidence across medication classes can identify knowledge gaps, which may pave the way for more integrated research on discontinuation.

## Introduction

Clinical guidelines generally recommend prolonged pharmacological treatment following symptomatic remission, for example for antidepressants [1, 2] and antipsychotics [3], even though the minimal duration of continuation is often unspecified or variable [4, 5]. Although prolonged medication use can be effective, most patients discontinue their medication at some point, sometimes due to poor insight, but also because of symptomatic remission, lack of effectiveness, adverse events, or a perceived negative impact on functioning [6, 7]. However, patients and physicians are also concerned about episode recurrence and possible discontinuation symptoms (DS) [8, 9]. Given the frequency of discontinuation in clinical practice and the possible consequences, it is essential to have evidence-based clinical information on rational discontinuation strategies to guide patients, clinicians and policymakers.

In psychiatry, discontinuing medication is relevant for all major medication classes, i.e., antidepressants, antipsychotics, benzodiazepines, mood stabilizers, stimulants, and opioids. This may either involve complete discontinuation (stopping) or dose reduction strategies (staying on medication at a lower dose, sometimes below the minimally effective dose) [10]. Although answers to discontinuation topics may differ between medication classes, there may also be similarities. Therefore, comparing and integrating evidence can help to establish similarities and differences regarding discontinuation. Summarizing existing discontinuation knowledge can further help provide answers for patients and physicians and optimize discontinuation care.

Here, we provide an overview of the current evidence on discontinuation for all major medication classes used in psychiatry. For an overview of the pharmacological mechanisms hypothesized to subtend discontinuation symptoms, we refer to the Supplementary Text and Supplementary Fig. 1. Opioids are included due to their clinical relevance in discontinuation care (both illicit and prescription opioids) within psychiatry. We further discuss three major clinical questions for each medication class: (1) Who can discontinue with an acceptable risk of recurrence or discontinuation problems? (i.e., determinants of risk of DS, and relapse and recurrence risks following discontinuation); (2) When is the best time to discontinue following symptomatic remission?; and (3) How can medication best be discontinued to prevent recurrence or DS? (i.e., dose reduction strategies and effective discontinuation interventions). Finally, we compare similarities and differences across the medication classes.

## Methods

The first and last authors revisited the current evidence on discontinuation across the six major medication classes used in psychiatry: antidepressants, antipsychotics, benzodiazepines, mood stabilizers, stimulants, and opioids. Experts from each medication class were invited to contribute for their respective field. All experts have made significant contributions to the literature in their respective field and most experts have multiple year of experience clinically guiding patients in discontinuing their psychotropic medication. Four consensus meetings were held between January 2021 and October 2021. During the consensus meetings, we defined three essential questions on discontinuation that we considered to be the most clinically relevant:

1. Who can discontinue with an acceptable risk of recurrence or discontinuation problems? (i.e., determinants of risk of DS and relapse and recurrence risk following discontinuation);
2. When is the best time to discontinue following remission? (i.e., optimal timing of discontinuation); and
3. How can medication be most effectively discontinued to prevent recurrence or DS? (i.e., dose reduction strategies and discontinuation interventions).

For each psychotropic medication class, one or several expert(s) then conducted a targeted literature search and selected high-quality key research articles in the literature related to their area of expertise. Key search terms were: “antidepressants”, “antipsychotics”, “mood stabilizers”, “benzodiazepines”, “stimulants”, “opioids”, “discontinuation”, “tapering”, “discontinuation symptoms”, “discontinuation syndrome”, “withdrawal”, and “relapse”. After examining the literature on discontinuation, the expert panel identified commonalities between medication classes and reached consensus with regards to recommendations for clinical practice as well as future research avenues. Finally, the first and last authors critically revised and integrated these contributions into the current piece.

## Discontinuation evidence across psychotropic medication classes

In the following section, we examine the three key clinical discontinuation questions (summarized in Table 1).

**Table 1:** Summary of the major discontinuation evidence across medication classes.

	Relapse/recurrence rates after discontinuation vs. continuation (%)	DS chances per cessation attempt (%)	When to discontinue	Specific drugs with discontinuation problems	Discontinuation interventions
Antidepressants	40% vs. 20% after 1 year [1, 2, 11–13]	27–86% [17]	For first-episode MDD: continue at least 4–6 months, longer after recurrent episodes [29, 31]	Paroxetine, duloxetine, and venlafaxine [17, 20, 156]	Preventive CBT [37, 38, 157]
Antipsychotics	64% vs. 25% after 1 year [3]	37–70% [47]	First episode of psychosis (FEP): continue at least 1–2 years (with rapid full remission and few risk factors, discontinuation after 6 months may be considered) [53]	Clozapine	Relapse prevention plan with patient and family [67]
Mood stabilizers	54% vs. 25% after 6 years [70]	Unknown	Continue maintenance treatment in already recurrent bipolar disorder for many years or even indefinitely [68]	Lithium; valproate	Relapse prevention plan with patient and family; frequent monitoring and continue monitoring for at least 1 year after discontinuation
Benzodiazepines	Largely unknown beyond benzodiazepine use itself	59–78% [84, 85]	Discontinue benzodiazepines as soon as possible [94]	Short half-life benzodiazepines (e.g., lorazepam, temazepam) [89–91, 158]	Pharmacological: valproate, pregabaline [93], carbamazepine, TCAs [83, 93] Psychological: CBT, minor interventions [87, 100]
Opioids	As analgesic: largely unknown As agonist treatment for illicit opioid use: 71% relapse into illicit opioid use	Unknown	As analgesic: discontinue opioids as soon as possible [132]. As agonist treatment: consider only if patient want to taper and risk of relapse into illicit opioid use is considered limited	Opioids with a short half-life (e.g., morphine, oxycodone), and opioids with high mu-receptor affinity (e.g., fentanyl) [121]	Pharmacological: Clonidine, anti-emetics, loperamide, lofexidine, guanfacine [137, 159] Rotation to long-acting agent to facilitate gradual tapering. Psychological: CBT. Mindfulness [138]
Stimulants	60–70% with re-emergent ADHD symptoms [141, 142]	<5% [120, 143]	Evaluate the use of stimulants annually [145]	N/A	Benzodiazepines [150]

### Antidepressants

#### Who can discontinue?

##### Relapse rates after discontinuation

Continuing antidepressants in patients with major depressive disorder (MDD) who responded to acute treatment halves the relapse risk compared to placebo (from 40 to 20%), in both specialized and primary care [1, 2, 11–14]. The most recent meta-analysis on this topic found that the number needed to treat (NNT) was 6, favouring continuation of antidepressants [15]. A landmark study pointed in the same direction in the context of primary care [16]. An important limitation of double-blind placebo-controlled discontinuation studies is their relatively short follow-up period (study duration averaging 40 weeks) and often rapid or abrupt antidepressant discontinuation, although the absolute relapse risk gradually decreases over time in discontinued patients [2, 15].

##### Discontinuation symptoms (DS)

DS of antidepressants include a range of somatic (e.g., nausea, dizziness) and psychological (e.g., irritability, low mood) symptoms [8], and are summarized using the FINISH acronym, which stands for Flu-like symptoms, Insomnia, Nausea, Imbalance, Sensory disturbances, and Hyperarousal (anxiety/agitation). DS in SSRIs were found to be higher in both surveys of selected populations and open-label studies [17, 18] (55%), than in double-blind studies (30%) [19], possibly due to smaller nocebo effects in double-blind studies and selection bias in surveys. DS especially occur during treatment with SSRIs and SNRIs that have relatively short elimination half-lives (<24 h), such as paroxetine and venlafaxine [17, 20], though other mechanisms unrelated to elimination contribute as well. For example, paroxetine has strong affinity for the M_1_ receptor and also inhibits its own metabolism (just like fluvoxamine and fluoxetine) [21]. Abrupt discontinuation leads to higher DS incidence rates (35–100%) compared to tapering over several weeks (42–56%) for paroxetine [17]. Conversely, fluoxetine has an elimination half-life of around 5 days (active metabolite 4–16 days), and is generally associated with fewer DS [22]. It is hypothesized that most DS coincide with the largest reductions in serotonin transporter (SERT) occupancy, which particularly occur at lower doses [8, 23, 24]. DS generally quickly resolve after dose increases or reinstatement of the antidepressant, which can aid in distinguishing DS from relapse [8].

##### Risk factors for relapse or DS following discontinuation

To our knowledge, based on a systematic review, no consistent empirical evidence links patient characteristics to predicting DS, including residual symptoms, number of prior episodes, and duration of antidepressant use [25]. Length of antidepressant use has been suggested to be a risk factor for some antidepressants, though the data are inconsistent [1]. Possible predictors of relapse following discontinuation are effort-reward decision time and cognitive reactivity, the latter being the most consistent predictor independent of residual symptoms [26–28]. However, these predictors have not been firmly established.

#### When to discontinue?

Most guidelines recommend to continue antidepressants for at least 4–6 months after successful acute-phase therapy in patients with first-episode MDD to prevent relapse, and longer periods after recurrent MDD [29–31]. Nevertheless, there is little evidence supporting a minimum duration of antidepressant treatment after stable remission. Patients with recurrent MDD should stay on treatment longer and sometimes indefinitely [16].

#### How to discontinue?

##### Discontinuation strategies

Consensus supports slow discontinuation of antidepressants, but few randomized studies have compared discontinuation strategies. Most studies employ a rather rapid 2-week tapering period with at most two steps [32–34]. Compared to abrupt discontinuation, 1–3-week tapering schedules lower DS frequency [32–34]. Although hyperbolic tapering, which involves decreasing relative dose reductions over time (Supplementary Text), is supported by expert opinion, it lacks evidence [8, 35]. Given the heterogeneity of DS between antidepressants and individual patient variation, general advice would be to slowly taper in those taking antidepressants for longer periods of time (e.g., years), and antidepressants with higher DS risk.

##### Dose reduction

A meta-analysis of five randomized trials found that dose reductions of phenelzine, maprotiline, imipramine, amineptine, and paroxetine increased the risk of MDD relapse by 62% relative to the group randomized to unchanged continuation [36].

##### Discontinuation interventions

A meta-analysis of four randomized controlled trials found no significant difference in time to depression relapse between patients receiving psychological interventions (e.g., cognitive behavioral therapy) during or after antidepressant tapering, compared to those who continued with antidepressant maintenance therapy only [37]. Another meta-analysis of 12 RCTs showed that MDD patients receiving psychotherapy during antidepressant treatment or tapering had a lower rate of relapse compared to those receiving active controls (antidepressant treatment) or treatment as usual [38]. In conclusion, a (preventive) psychotherapy is as effective as antidepressant maintenance.

### Antipsychotics

#### Who can discontinue

##### Relapse rates after discontinuation

Within 18 months after treatment initiation, 74% of patients with schizophrenia discontinue because of adverse events or insufficient efficacy [39]. A comprehensive network meta-analysis of RCTs found that all continuation strategies outperformed discontinuation, with large risk reductions for continuing at standard dose (RR = 0.37; NNT = 3.2) and switching antipsychotics (RR = 0.47; NNT = 3.6) [40]. Dose reduction was moderately effective in relapse prevention (RR = 0.68; NNT 6.3) but was outperformed by continuation and switching strategies [40]. Doses below recommended levels were also associated with relapse in other studies (in first and multi-episode schizophrenia), possibly due to insufficient D_2_ receptor occupancy [41, 42]. Moreover, risk of poor clinical outcomes doubles in schizophrenia patients discontinuing antipsychotics at 10-year follow up relative to those continuing antipsychotics [43]. This is supported by European and Asian registry observational and cohort studies, which found lower rehospitalization rates in patients continuing antipsychotics compared to discontinuation [44]. It is also supported by observed reductions in relapse rates and mortality in patients on long-acting antipsychotics as compared to those taking oral antipsychotics [45, 46].

##### Discontinuation symptoms (DS)

Antipsychotic-associated DS can be somatic (e.g., nausea, sweating), motor (e.g., restlessness), and psychological (e.g., irritability) in nature [47]. Hyperkinesia may also occur upon withdrawal and can last for months [48]. A systematic review of five studies (of mediocre quality) found a pooled frequency of DS of 53%, while abrupt discontinuation resulted in DS in 37–70% of patients (Table 1), with nocebo effects potentially playing a role [47].

##### Risk factors for relapse following discontinuation

Established risk factors for relapse after discontinuation or dose reduction include a diagnosis of schizophrenia (as compared to other psychotic disorders), long illness duration, poor pre-morbid functioning, no receipt of psychosocial intervention programs during discontinuation, shorter follow-up and in-hospital care [49–51]. Increased prolactin concentration, recurring hospitalizations, higher score on the Clinical Global Impression (CGI) severity scale, smoking, oral antipsychotic treatment (compared to long-acting injectables) and smoking have also been identified as predictors for relapse after discontinuation [52].

#### When to discontinue

Even though guidelines differ in their recommendations, several of them advise antipsychotic continuation up to 1–2 years after a first episode of psychosis (FEP) and life-long maintenance treatment after multiple psychotic episodes [53]. For those with a FEP, quick recovery and few poor prognostic factors, discontinuation may be considered after 6 months of remission. Relapse risk increases with longer illness duration and time since discontinuation [43, 54, 55]. We therefore emphasize the need for careful shared decision-making on discontinuing antipsychotics for patients who have been using them for longer time periods.

#### How to discontinue

##### Discontinuation strategies

For antipsychotic discontinuation, no evidence exists that gradual tapering decreases the risk of DS, although some recommend gradual and slowly (in months, possibly hyperbolic) tapering over quicker or abrupt discontinuation (Supplementary Text) [51, 56]. Tapered discontinuation (compared to abrupt discontinuation) may reduce the risk of adverse somatic events after full discontinuation [57], although the minimum dose threshold appears to have more relevance [51].

##### Dose reduction

To our knowledge, no published trials have compared dose reduction with complete discontinuation. A meta-analysis found that dose reduction doubles relapse risk relative to dose maintenance, particularly below 200 mg/day chlorpromazine equivalent or 2.5 mg risperidone equivalent, highlighting the importance of the extent of dose reduction [58, 59]. The one trial at the time of writing that had compared antipsychotic dose reduction with maintenance strategies was conducted in people with recurrent psychotic disorders and FEP [10]. It compared maintenance treatment with gradual, monitored, reduction of antipsychotics over several months (median dose reduction at any point 67% in the reduction group and 0% in the maintenance group). The results indicate that social functioning after 2 years is equal between groups, but that the risk of severe relapse is higher in the dose reduction than the maintenance group [10]. In contrast, observational data hint at possible functional improvements in functioning after antipsychotic dose reduction in similar patient populations [60, 61], but the possibility of confounding by indication precludes definitive conclusions from such data. New trials are underway to compare dose reduction or drug discontinuation with continuation strategies [62, 63], including guided dose reduction to establish the minimum effective dose [64]. Besides dose reduction studies, intermittent dosing strategies have also been proposed, with either regular gaps in dosing (from alternate days to several weeks) [65], or only initiating antipsychotics when psychotic symptoms emerge, with no antipsychotic use between psychotic episodes. While the first strategy has shown some merits if drug-free days are few, the second strategy increased relapse risk and rehospitalization [66], limiting the current role of intermittent dosing strategies for antipsychotics.

##### Discontinuation interventions

No interventions to mitigate relapse risks in those discontinuing antipsychotics have been described. In clinical practice, patients tapering antipsychotics are closely monitored, family members may be involved, and psychosocial interventions may be considered [67].

### Mood stabilizers

#### Who can discontinue?

##### Relapse rates after discontinuation

General: the question whether to continue or stop a mood stabilizer (lithium, valproate, lamotrigine, and carbamazepine) is relevant in long-term prophylactic (maintenance) treatment of bipolar disorder [68]. Patients with multiple recurrences are obvious candidates for long-term maintenance pharmacotherapy, but convincing evidence for this strategy is scarce [69]. Sustained (e.g., ≥10 years) maintenance therapy without relapses can hint that treatment may be no longer needed, but can also indicate treatment effectiveness, given the general assumption that bipolar disorder is a lifelong illness with a high risk of recurrence [68]. Given the highly heterogeneous course of bipolar disorder, decisions about whether or not to discontinue maintenance treatment require a thorough evaluation of illness course before and during treatment and assessment of risk factors for recurrence in each individual patient, as well as patients previous experiences with stopping mood stabilizers [68].

Lithium: few studies have reported follow-up after discontinuation vs. discontinuation of maintenance treatment. One observational study found that continuous lithium use reduced relapse risk by half compared to discontinuation during 6 year follow-up [70]. It is important to note that lithium discontinuation, particularly abrupt discontinuation, can lead to increased suicidality [71].

Valproate/carbamazepine/lamotrigine: a meta-analysis on lithium, lamotrigine, and second-generation antipsychotics (SGA’s) found a 61% relative risk reduction after 6 months in continuation compared to discontinuation groups [72].

Antipsychotics: although SGA’s are not mood stabilizers in the strict sense, several SGA’s have been recommended for maintenance treatment in bipolar disorder as monotherapy or combined with a mood stabilizer: quetiapine, asenapine, aripiprazole, olanzapine, risperidone, paliperidone, and lurasidone [72].

##### Discontinuation symptoms (DS)

Lithium: discontinuation of lithium may lead to irritability, anxiety, restlessness, vertigo, dizziness, and lightheadedness, particularly in the first week. Symptoms are generally mild and self-limiting [48, 73]. Abruptly or rapidly stopping lithium (within 2 weeks) can lead to “rebound” manic and depressive episodes [74]. There is no strong evidence that lithium is less effective when discontinued and restarted, compared to continuous treatment, a phenomenon that has been seen in anecdotal case reports and in about 15% of patients across seven published cohort studies [75].

Valproate/carbamazepine/lamotrigine: for carbamazepine, valproate, and lamotrigine, discontinuation phenomena include anxiety, agitation, irritability, lack of energy, depression, insomnia, depersonalization, and impaired concentration and memory [48].

Antipsychotics: SGA’s have been addressed in the section on antipsychotics above.

##### Risk factors for relapse following discontinuation

In bipolar disorder, young age of onset, psychotic features, numerous previous episodes, rapid cycling, comorbid anxiety, comorbid substance use, and persistent subthreshold symptoms are risk factors for recurrence, even during maintenance treatment [76]. These factors likely increase the risk of relapse following discontinuation, although direct evidence is lacking.

#### When to discontinue?

Most guidelines recommend that mood stabilizers for acute manic or depressive episodes be continued at least for several months after symptomatic remission (i.e., during the continuation treatment phase following the acute treatment and preceding maintenance treatment) [77], but evidence is scarce. However, in most patients with bipolar disorder it is recommended that a mood stabilizer should be continued for long-term maintenance treatment to prevent recurrences and improve inter-episodic functioning [68, 76].

#### How to discontinue?

##### Discontinuation strategies

Lithium: gradual discontinuation of lithium (at least over a month) reduces risk of DS and early recurrence relative to rapid discontinuation [74]. In clinical practice, lithium and other mood stabilizers are often tapered hyperbolically (Supplementary Text) [78]. If lithium needs to be discontinued because of inefficacy or severe side effects, it may be replaced with another mood stabilizer or SGA [79].

Valproate/carbamazepine/lamotrigine: it is unclear whether this also applies to other mood stabilizers [48].

##### Dose reduction

Lithium: for lithium, recommended effective doses for the prevention of manic and depressive episodes result in blood plasma levels of 0.6–0.8 mmol/l, or, in some cases, 0.4–0.6 mmol/l [80]. Dose reductions below these levels may diminish effectiveness.

Valproate/carbamazepine/lamotrigine: minimally effective blood levels are less robustly established than for lithium: 50 mg/l for valproate, 4 mg/l for carbamazepine, and 3 mg/l for lamotrigine.

##### Discontinuation interventions

There are no known interventions to reduce relapse risk or DS during or after mood stabilizer discontinuation. Closely monitoring mood symptoms during discontinuation of mood stabilizers is recommended, for at least 1 year [78].

### Benzodiazepines

#### Who can discontinue?

##### Relapse rates after discontinuation

Relapse rates for benzodiazepines are generally intricate to ascertain given the diversity of symptoms for which benzodiazepines are prescribed. Nevertheless, the majority of patients succeed in discontinuing benzodiazepines, as shown by systematic reviews and meta-analyses that found an average 1-year discontinuation success rate of 55–64% [81–83].

##### Discontinuation symptoms (DS)

Most patients discontinuing benzodiazepines experience some degree of DS (59–78%) [84, 85]. Physical DS of benzodiazepine discontinuation include muscle tension, weakness, spasms, pain, and influenza-like symptoms (sweating and shivering). Psychological DS include anxiety and panic attacks, restlessness, agitation, depression, mood swings, concentration difficulties, and sleep disturbances [86, 87]. In rare instances, rapid discontinuation may induce more severe DS, including seizures and psychotic symptoms [88]. DS may emerge or worsen weeks after discontinuation, with highest risks in short elimination half-life benzodiazepines (temazepam and lorazepam) [89–91].

##### Risk factors for relapse following discontinuation

Patients often want to stop after long-term benzodiazepine use, but face barriers such as dependence and misuse, reliance on benzodiazepines for comfort, and anticipation of sleep problems [92]. Intrinsic motivation and sufficient time to consider the benefits of discontinuation can help [92].

#### When to discontinue?

For most patients, adverse events of long-term benzodiazepine use (e.g., increased fall risk, sleepiness and cognitive impairment) do not outweigh the benefits [93]. For the sedative and anticonvulsant actions of benzodiazepines, discontinuation should commence before tolerance develops (i.e., first weeks of use) [94]. There is no evidence suggesting that discontinuation is easier after short-term use than long-term use. Regarding the anxiolytic and amnesic effects of benzodiazepines, there is no evidence that tolerance develops. The risk/benefit ratio of continued use should be discussed at least annually and discontinuation advised where possible.

#### How to discontinue?

##### Discontinuation strategies

Benzodiazepines should be gradually discontinued to prevent seizures and severe DS after more than 3 months of use [78]. Even after chronic use, discontinuation over a period of less than 6 months is feasible and appropriate [95]. Gradual dose reduction reduces DS intensity and improves discontinuation success [95]. However, no RCTs have compared different dose reduction methods. UK guidelines pragmatically suggest a reduction of about 12.5% of the daily dose every 2 weeks [96]. In clinical practice, a weekly reduction of 25% for the first 75% of the dose, followed by hyperbolic tapering or steps of 12.5% for the final 25% of the dose is often used [97]. There is no evidence for week-on week-off (pulse) dosing [98]. Switching to a long-acting benzodiazepine before discontinuation may be preferable for some patients, particularly when discontinuation attempts have failed, or with high doses (over 30 mg diazepam equivalent) [90, 95, 99]

##### Dose reduction

No evidence comparing dose reduction to discontinuation exists. Long-term use of benzodiazepines is generally not recommended and complete discontinuation should be attempted.

##### Discontinuation interventions

Cognitive behavioral therapy add-on results in little added value over solely gradual dose reduction in the long run (>6 months post-intervention) [87, 100]. Multifaceted deprescribing interventions are effective in reducing benzodiazepine use [101]. While motivational interviewing may be used, there is insufficient evidence for its standard application [87]. Gradual dose reduction combined with a brief or “minimal” intervention (personalized letter, self-help information) in primary care is superior to routine care alone [102–104]. Generally, interventions focused on patient self-awareness and education up discontinuation successes [92]. Valproic acid, carbamazepine and tricyclic antidepressants may help with benzodiazepine discontinuation, albeit with low quality evidence [83, 93]. The use of flumazenil for benzodiazepine discontinuation remains experimental [105, 106].

### Opioids

#### Who can discontinue?

##### Relapse rates after discontinuation

Opioids are commonly prescribed for two indications: analgesic indications and agonist treatment in patients with illicit opioid use. As an analgesic, opioids have a place in acute and cancer-related pain. However, continued long-term use for chronic non-cancer pain may induce adverse events, such as elevated pain, mood disorders and reduced quality of life, without clear benefits as an analgesic [107–111]. In these cases, opioid discontinuation should be initiated as early as possible as nearly 5% of patients develop an opioid use disorder [112]. Patients with chronic non-cancer pain and an opioid use disorder discontinuing their opioids have a relapse rate of 23% after 1 year [113, 114]. In patients on opioid agonist treatment (methadone or buprenorphine), the aim is to reduce opioid-related harm [115]. These patients generally had several previous failed attempts to stop illicit opioid use. Patients with illicit opioid use disorder who use agonist treatment have a relapse rate of 71% within 1 month after discontinuation of agonist treatment [113, 114]. Treatment goals for these patients should be more toward harm reduction with opioid agonist therapy (i.e., methadone or buprenorphine) instead of tapering. Only in a subset of individuals who have a wish to taper or discontinue their agonist treatment, and have improved psychological or social conditions, such as improvement on co-occurring mental disorders or housing situations, tapering or discontinuation can be considered [115].

##### Discontinuation symptoms (DS)

Opioid discontinuation in patients with chronic pain reduces pain severity and improves quality of life [116]. Increased pain and craving can occur in some patients in short term, particularly at higher doses [117]. In patients on agonist treatment, discontinuation of agonist treatment has been associated with increased craving levels, and reduced quality of life. Discontinuation of prescription opioids is usually accompanied by DS after more than 3 weeks of use [116, 118, 119]. Typical opioid DS include dysphoria, anxiety, agitation, nausea, muscle ache, rhinorrhea, sweating, diarrhea, yawning, fever, and insomnia [120]. Higher opioid doses and long-term use result in more severe DS, while long-acting opioids (methadone or buprenorphine) cause less severe DS [121]. Among patients with opioid use disorders, acute discontinuation or rapid tapering of opioids may lead to withdrawal that requires medical care [122]. In individuals discontinuing opioids after long-term use for chronic pain, suicidal ideation and self-directed violence may occur [123,124]. Overdose-related morbidity is more likely in the first month after discontinuation, due to decreased tolerance to original doses following relapse [125]. Opioid discontinuation may result in illicit opioid use, including heroin and illegally manufactured fentanyl [126].

##### Risk factors for relapse following discontinuation

Individuals dissatisfied with their opioid use and those not experiencing the desired analgesic effect are more likely to discontinue opioids successfully [118, 127]. Young age, low doses, short duration of use, good socio-economic context, and a negative history of psychiatric illness are positively associated with successful opioid discontinuation [118, 127–130]. Conversely, adequate depression treatment may increase the incidence of opioid use cessation in patients with chronic pain [131].

#### When to discontinue?

Opioids prescribed as analgesics should be discontinued before tolerance develops as long-term use is not indicated for chronic (non-cancer related) pain [132]. The risk/benefit ratio of continued use should be discussed regularly and discontinuation advised when possible.

In patients on opioid agonis treatment, treatment effectiveness, and co-use of other drugs (including illicit opioids) should regularly assessed. In individuals who have a wish to taper or discontinue their agonist treatment, and have improved psychological or social conditions, such as improvement on co-occurring mental disorders or housing situations, tapering or discontinuation can be considered [115].

#### How to discontinue?

##### Discontinuation strategies

Patients on low-dose prescription opioids (<20 mg oral morphine equivalents) and/or for a short duration (<2–3 weeks) can generally discontinue without tapering [128]. In other patients, tapering is advised [128]. More rapid tapering generally results in more severe withdrawal and lower success rates [133, 134]. The advised rate of discontinuation in guidelines ranges between 10% per month and 50% per week of the initial dose, with slower tapers (i.e., 10% reduction per month) being more likely to be better tolerated [133, 134]. If rapid discontinuation of opioids is necessary for medical reasons, reduction strategies of up to 50% per week are possible [128]. Switching to long-acting opioids (usually 70% dose-equivalent) can be considered with high doses (>90 mg morphine equivalents) and/or prolonged opioid use (>3 months), but also in patients with prior failed discontinuation attempts. Long-acting opioids should be tapered to the lowest possible dose, and then discontinued [135, 136].

##### Dose reduction

Discontinuation should be the primary goal in patients using opioids for analgesic purposes. Nevertheless, in patients at high risk of relapse (high opioid doses, long-time use, either with or without opioid use disorder) [118, 128, 129], long-acting opioid agonist treatment (e.g., methadone or buprenorphine) can be considered [137]. In patients on agonist treatment, dose reduction strategies are recommended when adverse events develop, or in the event of pharmacokinetic changes, e.g., due to co-medication, successful treatment of hepatitis, or ageing.

##### Discontinuation interventions

Mindfulness and cognitive behavioral therapy in patients with opioid use disorder result in lower perceived pain and focus on analgesics [138]. Such strategies may facilitate discontinuation of opioids, although evidence is lacking. There is limited evidence for clonidine for agitation, shaking and muscle ache, anti-emetics for nausea, and loperamide for diarrhea. For insomnia, benzodiazepines should be given with caution in this patient group, due to their risk of developing sedative use disorder [137].

### Stimulants

#### Who can discontinue?

##### Relapse rates after discontinuation

Patients with ADHD who continue stimulants have a better quality of life, less impulsive behavior and hyperactivity, compared to those who discontinue [139, 140]. The majority of patients who discontinue stimulants need to re-start treatment within 2 weeks due to worsening of ADHD symptoms REF. Nevertheless, ~30% of children with ADHD can discontinue their stimulants without worsening of ADHD symptoms at 3 months follow up [141, 142].

##### Discontinuation symptoms (DS)

DS are uncommon after discontinuation, although transient fatigue, insomnia (or hypersomnia), vivid dreaming, and irritability have been described [120, 143]. Transient motor symptoms may particularly occur with concomitant use of antipsychotics.

##### Risk factors for relapse following discontinuation

Re-emergence of ADHD symptoms is independent of age and sex, although adults may be at less risk compared to minors after stimulant discontinuation [139, 144]. There are no other reliable predictors for successful stimulant discontinuation.

#### When to discontinue?

Guidelines recommend to annually review patients taking prescription stimulants. During such evaluations, patients’ perspectives, risk-to-benefit ratios, and medication doses should be evaluated [145]. Prolonged treatment is recommended in adolescence, due to a high level of negative outcomes in ADHD patients during this age phase. The American Academy of Child and Adolescent Psychiatry suggests clinicians consider stimulant discontinuation in ADHD after a 1-year period without significant ADHD symptoms, dose adjustments, or symptom worsening during drug holidays [146].

#### How to discontinue?

##### Discontinuation strategies

Discontinuation of prescribed stimulants is considered safe, usually without tapering. In clinical practice, tapering stimulants is sometimes considered, while lacking evidence [139, 147].

##### Dose reduction

One RCT found that a 50% dose reduction results in significant worsening of ADHD symptoms [148]. At the risk of unwanted worsening drug adherence [149], drug-free “holidays” may have positive effects on ADHD symptoms and adverse events [139].

##### Discontinuation interventions

Guidelines on detoxification of non-prescribed stimulants support the use of supportive measures. Short-term (<2 weeks) use of benzodiazepines may reduce irritability and improve sleep [150].

### Integration of evidence across medication classes

From the current evidence across medication classes in this perspective, we identified seven commonalities that are discussed below (see also Supplementary Text, Section 2 for more details). An important limitation that should be kept in mind is that the discontinuation studies at hand often have methodological shortcomings, including short follow up, as well as abrupt or very rapid discontinuation schedules [151]. There is thus a strong need for more robust evidence to guide evidence-based decision-making on psychotropic drug discontinuation in clinical practice.

#### Discontinuation symptoms are common but remain difficult to predict

First, discontinuation symptoms are common, particularly after abrupt or rapid cessation of medication, except in the case of stimulants. These symptoms generally dampen over time and seem less likely when discontinuation occurs gradually. In general, DS risk is higher for psychotropic drugs with shorter elimination half-lives, such as paroxetine, venlafaxine, clozapine, morphine, oxycodone, lorazepam, and temazepam (Table 1). Nevertheless, estimations of DS prevalence across medication groups are heterogeneous (see also Table 1). A challenge is that DS are often not measured at all, and, if measured, their operationalization varies (e.g., concerning questionnaires, cutoff values, and time points) [18]. Moreover, in many blinded studies, medication is abruptly stopped. Thus, current estimations of DS not only reflect the situation following abrupt or very quick cessation, but also include the influence of expectations and patients and clinicians’ experiences and may thus be subject to nocebo effects. Finally, it can be challenging to disentangle DS from relapse due to symptom overlap [8]. This may result in DS being misdiagnosed as relapse in discontinuation studies [22].

#### Do not discontinue medication too quickly

Second, it is clear that abrupt discontinuation is not only associated with DS, but also entails increased relapse risks. Nevertheless, the optimal period during which medication should be discontinued and the rate of dose reduction are unclear. Pragmatically, discontinuation over months is advisable, but evidence for these strategies is lacking. Gradual lowering of the dose is generally recommended for several reasons. First, relapse may be less severe if a patient is on a medium or low dose relative to discontinuation. Second, steady-state may be more readily re-achieved if relapse occurs while a patient is on a lower dose. Third, gradual tapering may be more comfortable for patients because of the decreased likelihood of DS. Gradual tapering is often possible using conventional dose regimens, and many patients can discontinue using these existing doses. Nevertheless, when discontinuation poses a greater challenge, a gradual decrease with low doses may be feasible. Another option that becomes apparent across medication classes is the possibility to switch to long-acting agents, e.g., diazepam, sustained-release buprenorphine and fluoxetine (although of the latter intervention only case reports are known). For some medications, e.g., opioids and antipsychotics, (demographic) variables have been associated with successful tapering, but given the lack of replicated findings these are not ready for translation into clinical practice.

#### Urgent need for more interventions to help patients successfully discontinue

Third, the literature on pharmacotherapeutic and psychological interventions to help patients start and finish tapering regiments is heterogeneous. For benzodiazepines, easy-to-use interventions, such as letters, are effective, but whether this extends to other medication classes is unknown. For preventive CBT, there is some evidence that it can help successfully discontinue antidepressants. More intervention studies are needed to assess whether time-intensive psychosocial interventions, CBT or simple interventions can help patients discontinue. For drugs that can lead to physical dependence (i.e., benzodiazepines and opioids), the need to discontinue is often relatively urgent and pressing.

#### Many patients can successfully discontinue

Fourth, while relapse risks increase after discontinuation (two-fold for antidepressants, stimulants and higher for antipsychotics), a (considerable) proportion of patients do not relapse. Conversely, a substantial proportion of patients still relapses while using medication. Therefore, the long-term perspective of discontinuation is that the risk-benefit ratio may shift for patients toward stopping their medication at some point, at least once. The weighing of risks vs. benefits related to discontinuation are personal and involve considerations about side effects, patient preferences, psychiatric history, and treatment efficacy. We believe that (dis)continuation should be periodically discussed with patients and family, and an emergency plan with relapse signs and subsequent advised actions can be helpful.

#### Chronic treatment with benzodiazepines and opioids should be discouraged

Fifth, discontinuing benzodiazepines and opioids is different from stopping antidepressants, mood stabilizers, or antipsychotics due to their potential for dependence after chronic use. The decision to discontinue should, in our opinion, be based on shared decision-making between patients and their families, physicians, and pharmacists. Shared decision-making can facilitate one to come up with the most viable strategy, which often includes considerations about relapse risk as well as other domains, such as quality of life and well-being.

#### Relapse is too narrow an outcome

Sixth, studies have predominantly focused on relapse risk, largely ignoring other outcomes relevant to patients, such as social functioning or subjective well-being. Moreover, there are varying definitions of relapse and remission both within and across different diagnostic entities, such as MDD, bipolar disorder, and schizophrenia. Therefore, even though phenotypes may be comparable, unique patterns and courses within a certain disorder necessitate uniform criteria for what constitutes relapse or remission. This heterogeneity of definitions hampers comparisons of relapse rates across disorders, and emphasize to include outcomes that are transdiagnostic and transcend relapse-based outcomes. Incorporating a transdiagnostic approach in future discontinuation studies, and including patient groups with conditions like schizoaffective disorder and bipolar depression, is crucial to broaden our understanding across various psychiatric disorders, highlighting the need for adaptive and individualized treatment protocols, regardless of specific diagnosis.

#### The need for shared decision making and integrated deprescribing

Shared decision-making is essential for the success of the discontinuation strategy, tailoring decisions to individual patient needs, abiding by informed consent procedures and empowering a patient’s autonomy, while at the same time carefully weighing in the harm/benefit ratio of discontinuation [152]. Moreover, the overall infrastructure of a county’s healthcare system is an important determinant of how discontinuing practices can be implemented. For instance, in a well-resourced healthcare system, there might be more opportunities for close monitoring, early intervention, and comprehensive support services, which can be pivotal in preventing relapse. This aspect is often under-discussed but is critical for understanding and improving treatment outcomes in mental health care. Indeed, rational deprescribing can benefit from interdisciplinary support and interdisciplinary collaboration, wherein physicians and pharmacists together discuss with patients to decide whether or not to discontinue [153].

## Conclusion

Cross-pollination across medication classes has helped us to identify, catalog, and advance our knowledge on medication discontinuation in psychiatry. For clinicians it is important to have background knowledge about this topic to be able to advise on the safest strategies for treatment discontinuation for patients considering discontinuation [154]. There is already quite some evidence that can be applied to clinical practice where patients often have limited help in the discontinuation of their medication. At the same time, it is also clear that both the quantity and quality of discontinuation evidence is not only heterogeneous, but also quite limited [155]. Therefore, in Box 1, we have summarized several recommendations on how to further advance our knowledge about discontinuation in psychiatry. We believe research priorities should be drawn toward deprescribing in psychiatry, aiming to help patients and clinicians discontinue responsibly and jointly.

Box 1 Recommendations for future research to advance our knowledge about discontinuation of psychotropic medication Recommendations

From the current evidence across medication classes in this perspective, we signal several recommendations for future research:

1. More double-blind placebo-controlled discontinuation studies are needed that compare different discontinuation strategies, including the efficacy of hyperbolic discontinuation strategies, for which there is currently limited evidence.
2. Experimental medicine studies, including molecular imaging studies, are necessary to examine the mechanisms of discontinuation, also regarding the putative hyperbolic discontinuation strategies.
3. The RCT is the most convincing, but not the only study design that can answer discontinuation questions. Cohort/case-control studies, qualitative studies on subjective experiences, single case study designs and real-world studies may all result in relevant insights.
4. There is an urgent need for more reliable predictors at the level of the individual patient or medication that related to relapse and DS. Therefore, across discontinuation studies, both intervention or clinical studies, consistent information should be collected on patient and medication characteristics (e.g., the number of past episodes, personality characteristics, and neurobiological correlates).
5. Extend outcomes beyond the narrow outcomes of relapse, and include (long-term) functional outcomes and well-being.
6. We should strive for discontinuation studies where patients are allowed with residual symptoms to enter, and not limit to one specific DSM diagnosis (e.g., MDD).
7. More clinical and real-world studies should compare dose reduction with complete discontinuation.
8. With regards to the (neuro)biological and psychological mechanisms of discontinuation symptoms, we recommend integrating placebo/nocebo effects, hyperbolic tapering strategies, and the role of expectations in future discontinuation studies.
9. Discontinuation symptoms should be measured consistently across studies. The DESS scale is a possible candidate for antidepressants, the OWS (opioid withdrawal scale) for opioids, and the BWSQ (benzodiazepine withdrawal scale) for benzodiazepines.

## Supplementary information

Supplementary Text

Supplementary Figure 1

Supplementary Figure 1 Legend
