# Supplementary material for: Discontinuation of psychotropic medication: a synthesis of evidence across medication classes
Source: Mol Psychiatry. 2024 Mar 19;29(8):2575–86. doi: 10.1038/s41380-024-02445-4 (PMC11412909; doi:10.1038/s41380-024-02445-4)
Supplement: Supplementary file 1 — Supplementary Text [file 41380_2024_2445_MOESM1_ESM.docx]

**Supplementary text**

**Section 1:** **Pharmacological mechanisms underlying discontinuation symptoms**

The rationale behind the gradual tapering of psychotropic drugs is that biological systems will have more time to adapt to reductions in available ligands, thereby reducing the intensity of withdrawal symptoms.^1^ During tapering, psychotropic drug plasma levels will decrease, and subsequently the occupancy of its target will decrease as well. A hyperbolic relationship exists between drug dose or plasma level and target occupancy during treatment.^2^ Dose reductions during linear tapering presumably result in increasingly large reductions in target occupancy with consequently increasingly severe withdrawal reactions. In recent years, the concept of hyperbolic tapering has gained attention and has been proposed for antidepressants and antipsychotics.^1,3^ The aim of hyperbolic tapering is to achieve a linear reduction of pharmacological effect. However, designing a rational tapering strategy is complicated by the kinetics of the drug-target interaction and the pharmacological receptor and transporter (“target”) profile.

Firstly, hyperbolic tapering implies a direct correlation and equilibrium between decreasing drug dose or plasma level and receptor occupancy.^1^ However, plasma kinetics are not necessarily representative of brain kinetics, as for example SSRI elimination from the brain can be much slower that its plasma clearance.^4^ Moreover, several preclinical and clinical studies indicate that SERT and D_2_ occupancies decline in a linear fashion, even after acute discontinuation.^5-7^ Secondly, a complicating factor is that psychotropic drugs differ in the number of targets bound at therapeutic doses. SSRIs and TCAs share a high affinity for the serotonin transporter (SERT), but TCAs additionally have high affinities for the norepinephrine transporter (NET) and muscarinic acetylcholine (M), histamine (H)1, and alpha adrenergic (α) receptors. Similarly, first and second-generation antipsychotics share affinity for the dopamine (D)2 receptor, but second-generation antipsychotics have high affinities for 5-HT2A-, M-, H1-, and α1-receptors as well. While main target occupancy is responsible for the clinical efficacy, e.g. SERT and NET in antidepressants and D_2_ in antipsychotics, off-target binding is implicated in side effects.^2,8,9^ Hyperbolic tapering focuses on the main drug target, but disregards declining occupancy of secondary targets that can result in withdrawal symptoms.^3,10^

Target occupancy profiles based on relative target affinities can illustrate the pharmacological effects during tapering. During hyperbolic tapering of citalopram, SERT occupancy decreases from 80% to 0%, with only marginal secondary target effects **(Figure 1A)**. During hyperbolic tapering of amitriptyline, SERT and NET occupancies decline comparably from 60-80% to 0%. However, in the final stages of tapering from a SERT occupancy of 40%, H1-, M- and α1-receptors are still occupied > 80%. Therefore, significant secondary target withdrawal effects are expected during the final stages of amitriptyline tapering. A similar conclusion is drawn when comparing D2 based hyperbolic tapering of haloperidol and quetiapine, due to high quetiapine secondary target affinities **(Figure 1B**). Thus, the practice of hyperbolic tapering is insufficiently justified by primary and secondary target occupancy during tapering and warrants future research, especially on the effects on off-target receptors.

**Section 2: Commonalities Across Medication Classes**

Several commonalities were found across the different medication classes. First, many patients are able to discontinue, regardless of the (abrupt) strategy used. Indeed, discontinuation-associated relapse rates are strikingly similar across the groups of agents studied here (Table 1), with continued use reducing relapse risk by around 50%. Second, the quantity and quality of discontinuation evidence is not only heterogeneous but also often quite limited. For example, it is generally unknown who can safely discontinue at what time point. This hampers identification of the substantial proportion of patients with favourable outcomes after discontinuation. Third, relapse risks seem to decrease with time elapsed after discontinuation, making discontinuation efforts more likely to succeed after a longer time period. Fourth, when there are predictors of successful discontinuation (in the case of antipsychotics), these are not ready for translation into clinical practice. Fifth, optimal discontinuation strategies remain largely unknown (Table 1). Finally, studies have predominantly focused on relapse risk, largely ignoring other outcomes relevant to patients such as social functioning or subjective well-being. It is clear that there are several major knowledge gaps. In box 1, we have summarized several recommendations how to further advance our knowledge related to discontinuation in psychiatry.

**References**

1. Horowitz MA, Taylor D. Tapering of SSRI treatment to mitigate withdrawal symptoms. *The lancet Psychiatry* 2019; **6**(6): 538-46.

2. Meyer JH, Wilson AA, Sagrati S, et al. Serotonin transporter occupancy of five selective serotonin reuptake inhibitors at different doses: an [11C]DASB positron emission tomography study. *Am J Psychiatry* 2004; **161**(5): 826-35.

3. Horowitz MA, Jauhar S, Natesan S, Murray RM, Taylor D. A Method for Tapering Antipsychotic Treatment That May Minimize the Risk of Relapse. *Schizophr Bull* 2021; **47**(4): 1116-29.

4. Strauss WL, Layton ME, Dager SR. Brain elimination half-life of fluvoxamine measured by 19F magnetic resonance spectroscopy. *Am J Psychiatry* 1998; **155**(3): 380-4.

5. Tauscher J, Jones C, Remington G, Zipursky RB, Kapur S. Significant dissociation of brain and plasma kinetics with antipsychotics. *Mol Psychiatry* 2002; **7**(3): 317-21.

6. Takano A, Suzuki K, Kosaka J, et al. A dose-finding study of duloxetine based on serotonin transporter occupancy. *Psychopharmacology (Berl)* 2006; **185**(3): 395-9.

7. Arakawa R, Tateno A, Kim W, Sakayori T, Ogawa K, Okubo Y. Time-course of serotonin transporter occupancy by single dose of three SSRIs in human brain: A positron emission tomography study with [(11)C]DASB. *Psychiatry Res Neuroimaging* 2016; **251**: 1-6.

8. Luo H, Richardson JS. A pharmacological comparison of citalopram, a bicyclic serotonin selective uptake inhibitor, with traditional tricyclic antidepressants. *Int Clin Psychopharmacol* 1993; **8**(1): 3-12.

9. Kroeze WK, Hufeisen SJ, Popadak BA, et al. H1-histamine receptor affinity predicts short-term weight gain for typical and atypical antipsychotic drugs. *Neuropsychopharmacology* 2003; **28**(3): 519-26.

10. Dilsaver SC, Greden JF, Snider RM. Antidepressant withdrawal syndromes: phenomenology and pathophysiology. *Int Clin Psychopharmacol* 1987; **2**(1): 1-19.
