# Supplementary figures and images for: Discontinuation of psychotropic medication: a synthesis of evidence across medication classes
Source: Mol Psychiatry. 2024 Mar 19;29(8):2575–86. doi: 10.1038/s41380-024-02445-4 (PMC11412909; doi:10.1038/s41380-024-02445-4)

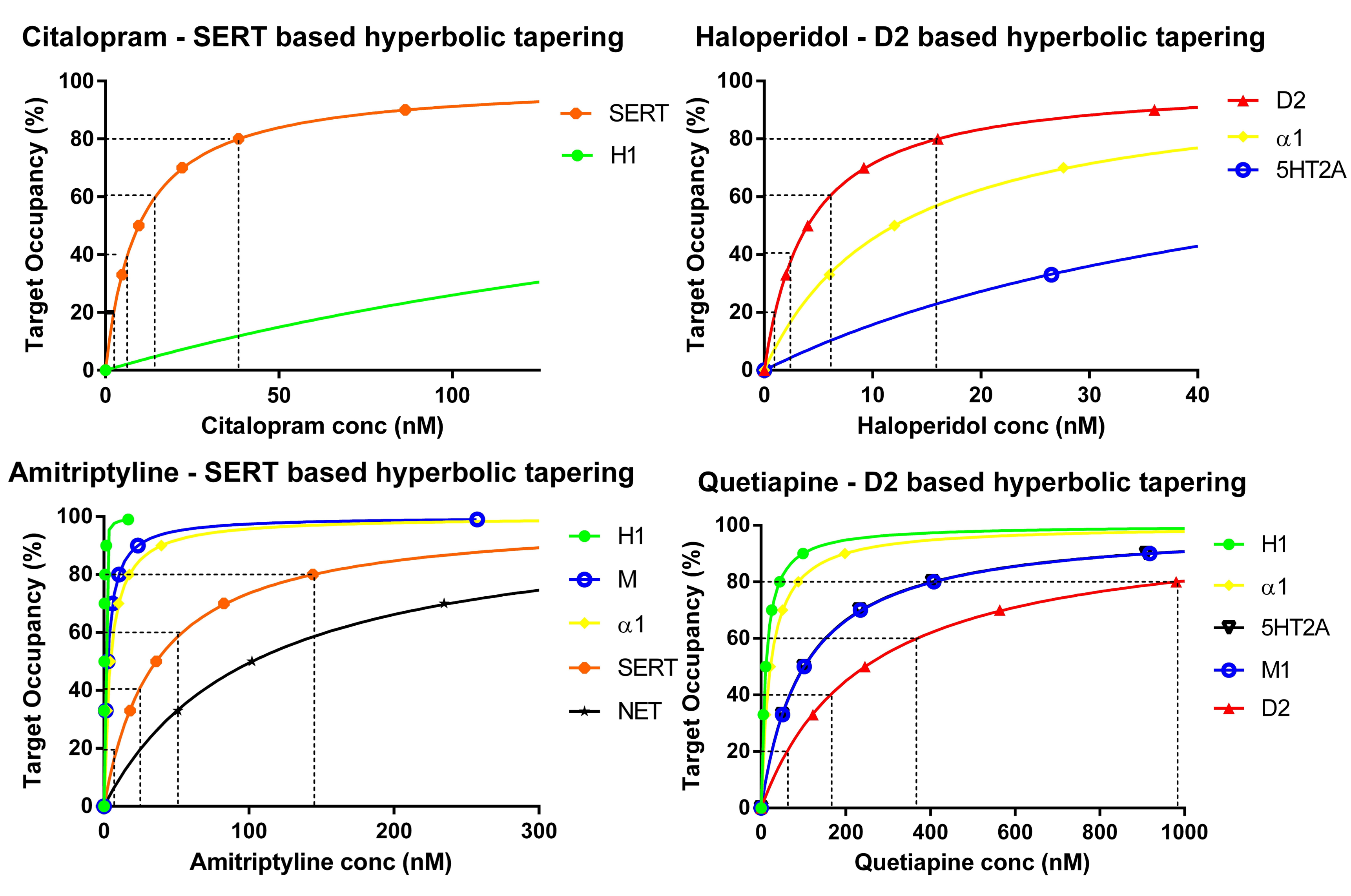

Supplement: Supplementary file 2 — Supplementary Figure 1 [file 41380_2024_2445_MOESM2_ESM.png]
