# Supplementary material for: Discontinuation of psychotropic medication: a synthesis of evidence across medication classes
Source: Mol Psychiatry. 2024 Mar 19;29(8):2575–86. doi: 10.1038/s41380-024-02445-4 (PMC11412909; doi:10.1038/s41380-024-02445-4)
Supplement: Supplementary file 3 — Supplementary Figure 1 Legend [file 41380_2024_2445_MOESM3_ESM.docx]

**Supplementary Figure 1:** Receptor occupancies following hyperbolic tapering of A) citalopram (upper left panel) versus amitriptyline (lower left panel); B) haloperidol (lower right panel) versus quetiapine (lower right panel). The target occupancy curves are based on the inhibition constants (Ki values) for the different target receptors for antidepressants and antipsychotics.
